# Differential Expression and Function of Stamp Family Proteins in Adipocyte Differentiation

**DOI:** 10.1371/journal.pone.0068249

**Published:** 2013-07-10

**Authors:** Jørgen Sikkeland, Fahri Saatcioglu

**Affiliations:** Department of Biosciences, University of Oslo, Postboks, Oslo, Norway; NIAID, United States of America

## Abstract

Six transmembrane protein of prostate (Stamp) proteins play an important role in prostate cancer cell growth. Recently, we found that Stamp2 has a critical role in the integration of inflammatory and metabolic signals in adipose tissue where it is highly expressed and regulated by nutritional and metabolic cues. In this study, we show that all *Stamp* family members are differentially regulated during adipogenesis: whereas *Stamp1* expression is significantly decreased upon differentiation, *Stamp2* expression is increased. In contrast, *Stamp3* expression is modestly changed in adipocytes compared to preadipocytes, and has a biphasic expression pattern during the course of differentiation. Suppression of *Stamp1* or *Stamp2* expression both led to inhibition of 3T3-L1 differentiation in concert with diminished expression of the key regulators of adipogenesis - CCAAT/enhancer binding protein alpha (C/ebpα) and peroxisome proliferator-activated receptor gamma (Pparγ). Upon *Stamp1* knockdown, mitotic clonal expansion was also inhibited. In contrast, *Stamp2* knockdown did not affect mitotic clonal expansion, but resulted in a marked decrease in superoxide production that is known to affect adipogenesis. These results suggest that Stamp1 and Stamp2 play critical roles in adipogenesis, but through different mechanisms.

## Introduction

Over the last decades, there has been a dramatic increase in the prevalence of obesity. A recent estimate indicated that more than 1.5 billion people world-wide are overweight or obese [1]. This is a consequence of imbalances in expenditure and intake of energy along with changes in nutrition sources [2]. Obesity is linked to an increased risk of developing various diseases such as type 2 diabetes, cardiovascular disease, hepatic steatosis, airway disease, neurodegeneration, biliary disease, and certain cancers [3]. These maladies are now among the leading causes of death worldwide [4].

The increase in obesity has focused attention on adipose tissue function and development. Adipogenesis, the process by which fibroblastic precursor cells or preadipocytes are converted into mature adipocytes, has been one of the most intensively studied model systems for cellular differentiation [5]. Most of the adipogenesis research has utilized pre-adipocyte cell culture models (e.g. the murine cell lines 3T3-L1 and 3T3-F442A) [6]. For 3T3-L1 cells, a hormonal mixture commonly containing dexamethasone, isobutylmethylxanthine and insulin is used to activate signaling pathways which initiate a cascade of transcription factors that drive the adipogenic program through the stages of mitotic clonal expansion, growth arrest, and terminal differentiation [7], [8]. The nuclear receptor Pparγ and members of the C/ebp family are critical determinants of this process together with an assembly of transcriptional co-regulators. More recently, new mechanisms and cellular processes regulating the adipogenic conversion have been reported (for a brief overview, see [9]). Of these, oxidative stress and reactive oxygen species (ROS) have been implicated in pre-adipocyte differentiation [10]. ROS can affect the preadipocytes as both an external or internal signal, and depending on the source and localization, it may either promote or inhibit differentiation in a given model system [11]–[16].

The Stamp family of proteins (also known as STEAPs) consists of three members (Stamp1-3) that share high sequence similarity in the putative six-transmembrane domain; a region homologous to F(420)H(2):NADP(+) oxidoreductases found in archaea and bacteria, as well as to the yeast FRE family of metalloreductases [17]. All Stamps have metalloreductase activity in HEK293T cells [18]. Furthermore, Stamp3 has been shown to be essential for normal iron metabolism in mice [19]. Stamp2 expression is induced by tumor necrosis factor alpha (TNFα) in 3T3-L1 cells (thus also called TNFα-induced adipose-related protein (Tiarp)) and its expression is increased during differentiation [20]. In addition, studies in knockout mice showed that Stamp2 integrates inflammatory and nutritional signaling in mice on a regular diet [21]. More recently, we have found that Stamp2 controls intermediary metabolites to regulate inflammatory responses and atherosclerosis in mice [22]. Human STAMP2 expression in human adipocytes is stimulated by TNFα and interleukin 6, and STAMP2 levels positively correlate with insulin sensitivity [23], [24]. Furthermore, recent human studies found STAMP2 expression decreased in obese and/or insulin resistant individuals [25]–[27]. These findings point to a protective role of Stamp2 in adipose tissue function in both human and mice. However, a recent report found that STAMP2 expression was increased in obese patients and this was linked to reduced insulin response in isolated adipocytes [28].

Here, we investigated the expression of the *Stamp* family during adipogenic conversion of 3T3-L1 cells, and show that they are differentially regulated during adipogenesis with distinct profiles. We also show that both *Stamp1* and *Stamp2* affect 3T3-L1 adipogenesis. Herein we explore the molecular details of this process.

## Materials and Methods

### Cell Lines and Cell Culture

3T3-L1 cell line (a generous gift from the lab of Gökhan S. Hotamisligil, ATCC, CL-173) was maintained in Dulbecco’s modified Eagle’s medium (DMEM) (Lonza) supplemented with 10% fetal bovine serum (FBS) (Saveen Werner), 50 U/ml penicillin-streptomycin (Lonza) and 2 mM L-Glutamine (Lonza) in a 5% CO_2_ humidified atmosphere. 3T3-L1 cells were differentiated to adipocytes by adding standard adipogenic cocktail (0.5 mM methylisobutylxanthine [Sigma-Aldrich], 5 µg/ml insulin [Sigma-Aldrich], and 1 µM dexamethasone [Sigma-Aldrich]) to post-confluent cells. 48 h later, cells were re-fed with normal growth medium containing 5 µg/ml insulin every second day until day 8. When indicated, 1 µM pioglitazone (Pio) (Sigma-Aldrich) was added to the adipogenic cocktail.

### Lentivirus Production and Establishment of Stable 3T3-L1 Cell Lines

pLKO.1 plasmids (Sigma-Aldrich) containing shRNA against *Stamp1* (sh-St1_1 [TRCN0000253445] and sh-St1_2 [TRCN0000253448]), *Stamp2* (sh-St2_1 [TRCN0000249066] and sh-St2_2 [TRCN0000249065]) or green fluorescent protein (*GFP*) (shGFP) were transfected together with a packaging plasmid (pCMV-ΔR8.2) and an envelope plasmid (pCMV-VGS-G) into HEK293T cells using Fugene 6 (Invitrogen). 48 h post transfection conditioned medium was harvested, filtered through a 0.45 µm filter (Millipore) and added to 3T3-L1 fibroblasts. 36 h post infection the 3T3-L1 cells were subjected to selection with 2 µg/ml puromycin for 7 days after which the cells were maintained in medium with 1 µg/ml puromycin. Unless specified, sh-St1_1 and sh-St2_1 cells were used in the experiments presented.

### Immunofluoresence Microscopy

3T3-L1 cells were plated on cover slips, grown to post confluency and treated with adipogenic cocktail for 16 h. Cells were washed briefly with phosphate buffered saline (PBS) and fixed in methanol at −20°C for 5 min. Cells were then blocked with 1% BSA (Sigma-Aldrich) for 30 min before incubation with C/ebpβ antiserum (1∶100) (Abcam, ab32358) at 4°C overnight and incubated with Alexa Fluor 488 goat anti-rabbit secondary antibodies (1∶500) (Invitrogen) for 1 h at room temperature. DAPI (Sigma-Aldrich) staining was used for visualizing the nuclei. Images were acquired with an Olympus FlowView FV1000.

### Oil Red O Staining

The cells were washed briefly with PBS and then fixed with 0.5% gluteraldehyde in PBS followed by washes with PBS and 60% isopropanol (Arcus) in PBS. The cells were then stained in Oil Red O solution (3 parts Oil Red O [0.5 g [Sigma-Aldrich] in 200 ml isopropanol] and 2 parts MQ water) for 15 min and washed with 60% isopropanol followed by a final wash in PBS. Images were taken with an AxioCam HRc (Zeiss). For quantification, the Oil Red O was extracted from the cells with 100% isopropanol for 5 min. The extracts were clarified by centrifugation at 10,000 g for 2 min and absorbance at 460 nm was determined with a multiplate reader (Victor2, PerkinElmer).

### Cell Counting

At indicated stages of differentiation, the cells were washed with PBS, dissociated with Trypsin EDTA (Lonza), diluted in DMEM and counted using a haemocytometer.

### NBT Assay

The cells were washed briefly with PBS and then incubated with 0.1 mg/ml Nitro Blue Tetrazolium (NBT) (Sigma-Aldrich) in PBS at 37°C for 90 min to allow blue formazan crystals to form. The cells were then washed with PBS and images were acquired with an AxioCam HRc (Zeiss). To quantify the formazan produced, the cells were lysed with a 2 M KOH/DMSO (Sigma-Aldrich) (1/1.17, v/v) solution for 15 min. The lysate was clarified by centrifugation at 10,000 g for 2 min and absorbance at 570 nm was determined with a multiplate reader (Victor2, PerkinElmer).

### Western Analysis

The cells were washed with PBS at the indicated time points and protein extracts were made in lysis buffer (20 mM HEPES [pH7.7], 0.3 M, NaCl 0.2 mM EDTA, 1.5 mM MgCl_2_, 1% Triton X-100, 0.1% SDS with 1X Protease inhibitor cocktail [Roche] and Phosphatase inhibitor cocktail [Roche]) for 1 h. 50–100 µg of protein extract was resolved on a 10% polyacrylamide-SDS gel, blotted to a PVDF membrane and incubated with antisera against C/ebpβ (1∶500) (Abcam, ab32358), C/ebpα (1∶500) (Santa Cruz, sc-61), Pparγ (1∶1000) (a generous gift from Professor H.I. Nebb, Santa Cruz, sc-7273), STEAP4 (1∶500) (Proteintech, 11944-1-AP) or β-actin (Sigma-Aldrich) (1∶10000) in 5% BSA (1% for STEAP4 antibody) in Tris buffered saline (TBS)-0.1% Tween. Images were obtained with a Kodak imaging station 4000R and the band intensities were determined using Carestream Imaging Software.

### Quantitative Reverse-transcription PCR (qRT-PCR)

Total RNA was extracted from cells using the Trizol reagent (Invitrogen). mRNA transcripts were converted to cDNA by the Superscript II (Invitrogen) reverse transcriptase using oligo(dT) primers (Sigma-Aldrich). cDNA was quantified by the Lightycler480 system using the SYBR Green dye (Roche). For each primer pair the crossing point (CP) values of a given PCR for a sample were set relative to the CP value of the wild type control group, while also correcting for primer specific reaction efficiency with an internal standard curve. The values were then normalized to the expression of the ribosomal gene *36B4*. All PCR products were analyzed by melting curve analysis. qRT-PCR primer sequences (all from Sigma-Aldrich) used in this study are as follows: *Stamp1,* forward 5′-ATA GGA AGT GGG GAT TTT GC-3′, reverse 5′-AGA TGT CTC AGG TCC CAC AA-3′; *Stamp2*, forward 5′- TCA CTT CCT TGC CAT CAG-3′, reverse 5′- GCT CCA CCT TAG AAT CGA AG-3′; *Stamp3*, forward 5′- CCG TCC ATT GCT AAT TCC CTC-3′, reverse 5′- CGG CAG GTA GAA CTT GTA GTG-3′; *aP2*: forward 5′-GTC ACC ATC CGG TCA GAG AG-3′, reverse 5′-TCG ACT TTC CAT CCC ACT TC-3′; *Pparγ*, forward 5′-GCC CTT TGG TGA CTT TAT GG-3′, reverse 5′-GGC GGT CTC CAC TGA GAA TG-3′; *C/ebpα*, forward 5′-GCG GCA AAG CCA AGA AGT C-3′, reverse 5′-GCG GTC ATT GTC ACT GGT CA-3′; *36B4*, forward 5′-AAG CGC GCG TCC TGG CAT TGT CT-3′, reverse 5′-CCG CAG GGG CAG CAG TGG T-3′.

### Statistics

Statistical analyses were performed using the Student’s t-test. Data are presented as means and error bars represent standard deviation. Significance was defined as p<0.05. All analyses were repeated with the Mann-Whitney-Wilcoxon (MWW) test, which resulted in the same outcome as obtained with the Student’s t-test.

## Results

### Regulation of Stamp Expression during 3T3-L1 Adipogenesis

**Figure 1:**
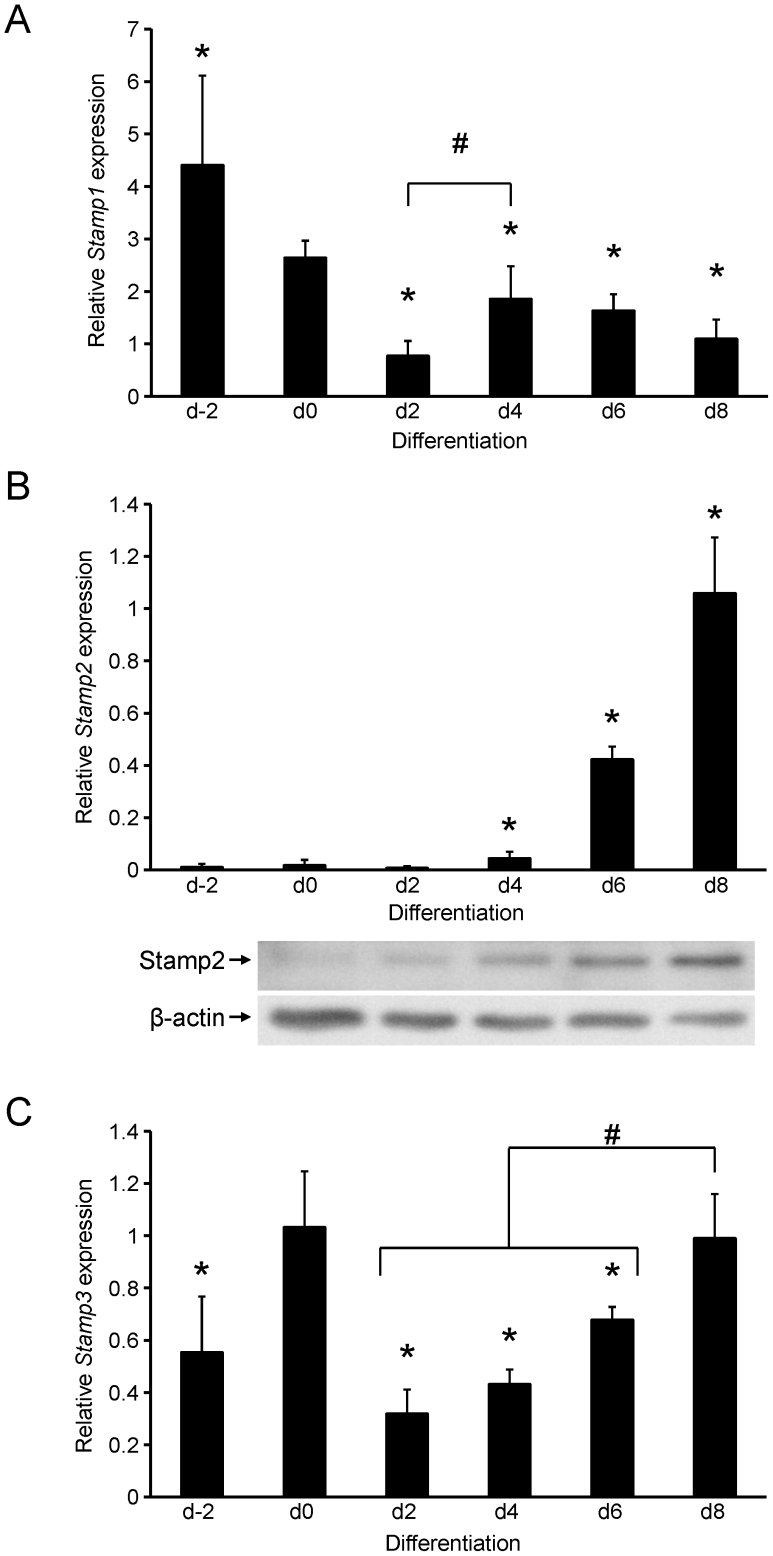
Regulation of Stamp family expression during 3T3-L1 adipogenesis. (A–C) qRT-PCR analysis of 3T3-L1 cells harvested at the indicated time points (days) of differentiation. The figures show the mRNA expression of *Stamp1* (A), *Stamp2* (B, top), and *Stamp3* (C) normalized to the reference gene *36B4*. The results are from three independent experiments, n = 9. *p<0.05 compared to d0; #p<0.05 between brackets. (B, bottom) Western analysis showing Stamp2 and β-actin protein levels from day 0 to day 8 of differentiation in 3T3-L1 cells harvested in parallel to those used for qRT-PCR analysis. The figure presented is representative of two independent experiments.

The increase in Stamp2 expression during 3T3-L1 differentiation into adipocytes has been reported previously [20], [21]. In addition, Stamp3 protein level was slightly increased upon 3T3-L1 differentiation and decreased in the adipose tissue of rats fed a high fat diet after a treatment with an anti-obesity drug [29], [30]. To investigate the coordinated expression of all Stamp family members in the same system, we differentiated 3T3-L1 fibroblasts into mature adipocytes and harvested proliferating cells (d-2), postconfluent cells (d0), and cells differentiated for 2, 4, 6 and 8 days (d2-d8), isolated RNA, and determined *Stamp* mRNA expression by qRT-PCR analysis (Figure 1). *Stamp1* mRNA expression was highest in proliferating 3T3-L1 fibroblasts (Figure 1A). Upon confluency, there was an approximately 40% reduction in *Stamp1* expression which decreased a further 70% at 48 h. Interestingly, the *Stamp1* expression rebounded at day 4 to about twice that of day 2 and then declined again during the rest of differentiation. Consistent with previous reports, *Stamp2* mRNA expression was low until day 4 when its expression increased to about 4-fold higher than in proliferating cells (Figure 1B, top). *Stamp2* levels increased dramatically after that reaching 60-fold higher levels by day 8 compared with what observed at day 0. Consistently, Stamp2 protein levels were barely detectable at day 0 and increased by approximately 30-fold by day 8 of differentiation (Figure 1B, bottom). In contrast to *Stamp1* and *Stamp2*, *Stamp3* expression showed a biphasic pattern rather than a distinct directional regulation pattern as it increased approximately 2-fold upon confluency, then dropped significantly to about 30% of this level by day 2, and then continued to rise again reaching similar levels of expression seen at confluency by day 8. Available antisera for Stamp1 and Stamp3 did not function in Western analysis and thus we were unable to explore relative changes to the protein levels for these proteins (data not shown). These data show that all Stamp family members are expressed in 3T3-L1 cells and are differentially regulated during adipogenesis.

### Knockdown of *Stamp1* and *Stamp2* Expression Suppresses 3T3-L1 Adipogenesis

**Figure 2:**
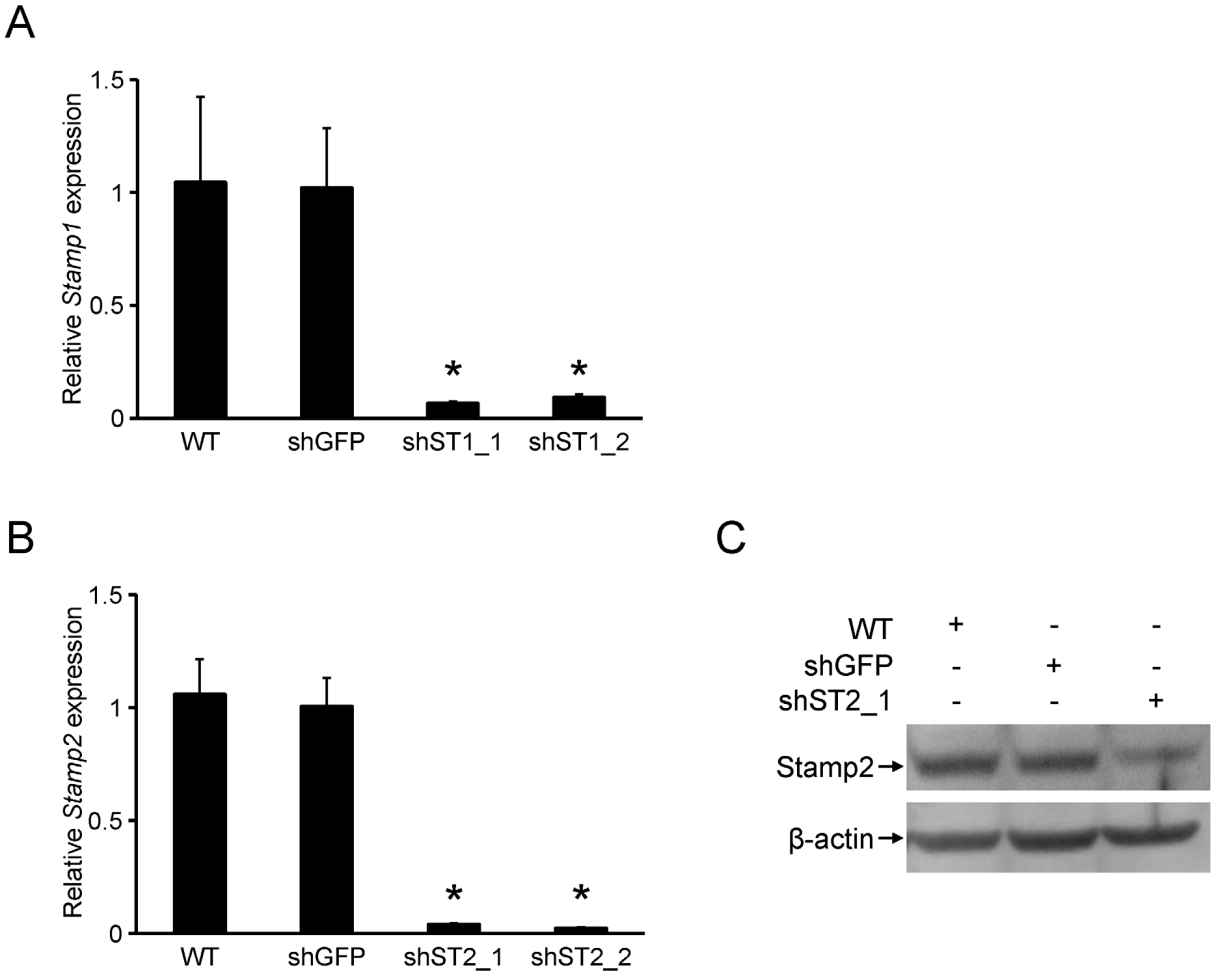
*Stamp1* or *Stamp2* knockdown in 3T3-L1 cells. (A–B) qRT-PCR analysis of WT, sh-GFP, sh-St1 and sh-St2 cells. The figures show relative mRNA expression of *Stamp1* (A) and *Stamp2* (B) normalized to the reference gene *36B4* from one experiment, n = 3. *p<0.05 compared to sh-GFP cells. The data presented are representative of three independent experiments. (C) Western analysis showing Stamp2 and β-actin protein levels at day 8 of differentiation in WT, sh-GFP and sh-St2 cells. The data presented are representative of two independent experiments.

Since *Stamp1* and *Stamp2* expression were most significantly affected during adipogenesis, we investigated the possible consequence of their knockdown on 3T3-L1 differentiation. To that end, we generated 3T3-L1 cell lines stably expressing short hairpin RNAs (shRNAs) against either *Stamp1* (sh-St1 cells) or *Stamp2* (sh-St2 cells), as well as Green Fluorescent Protein *(GFP)* (sh-GFP cells). There was an approximately 90% and 95% knockdown, respectively, of *Stamp1* and *Stamp2* expression, in these cell lines compared to sh-GFP cells (Figures 2A and 2B). Moreover, Western analysis showed that Stamp2 protein levels were reduced by about 70% in sh-St2 adipocytes compared to that of sh-GFP expressing or WT cells (Figure 2C).

**Figure 3:**
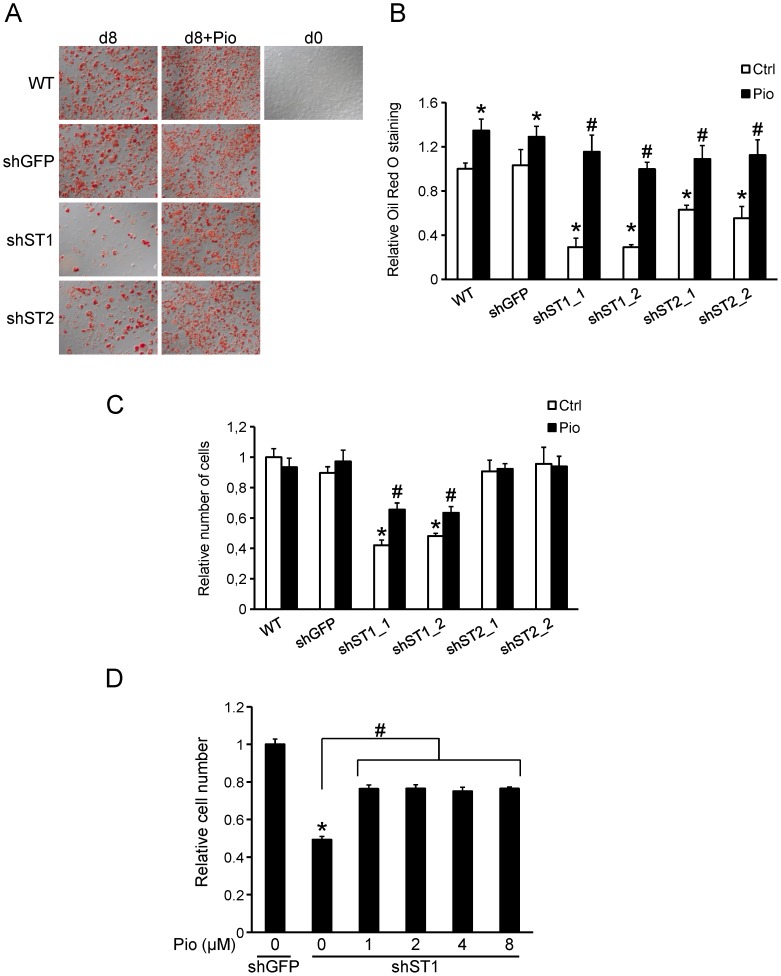
*Stamp1* or *Stamp2* knockdown reduces 3T3-L1 adipogenesis. (A–B) Oil Red O staining of WT, sh-GFP, sh-St1 and sh-St2 cells differentiated with pioglitazone (Pio) or vehicle (Ctrl). (A) Representative images of the staining. (B) Quantification from three independent experiments, n = 9. *p<0.05 compared to sh-GFP cells; #p<0.05 between Ctrl and Pio groups. (C) Relative cell number of the same cells as in (A). The results are from three independent experiments, n = 9. *p<0.05 compared to sh-GFP cells; #p<0.05 between Ctrl and Pio groups. (D) Relative cell number of sh-St1 cells differentiated with increasing concentrations of Pio. The results are from one experiment, n = 3. *p<0.05 compared to shGFP; #p<0.05 between brackets.

To assess the possible functional effect of *Stamp1* and *Stamp2* knockdown on 3T3-L1 cells, we differentiated sh-St1, sh-St2, sh-GFP and the wild type (WT) cells into adipocytes in the presence or absence of pioglitazone (Pio), an agonist of Pparγ and a known promoter of adipogenesis [31]. The cells were stained for lipid accumulation with Oil Red O to determine the extent of differentiation. The WT and sh-GFP cells showed similar levels of adipogenic conversion, distinctly increased compared to undifferentiated WT cells (Figures 3A and 3B). Differentiation in the presence of Pio further increased adipogenesis for WT and sh-GFP cells with 30% and 35%, respectively, consistent with previous findings [32]. sh-St1 and sh-St2 cells had significantly lower levels of differentiation (70% and 40%, respectively) in regular differentiation medium compared with the WT and sh-GFP control cells (Figures 3A and 3B). However, in the presence of Pio, this defect was rescued and the differentiation of sh-St1 and sh-St2 cells was comparable to that of WT and sh-GFP cells. These data show that *Stamp1* or *Stamp2* knockdown impairs adipogenesis and that this may be due to disrupted Pparγ signaling.

We have previously shown that STAMP1 and STAMP2 both increase human prostate cancer cell proliferation [33]–[35]. Since both *Stamp1* and *Stamp2* are differentially regulated during 3T3-L1 adipogenesis (Figures 1A and 1B), we examined if silenced expression of either would 3T3-L1 cell proliferation. To that end, equal numbers of WT, sh-GFP, sh-St1 and sh-St2 cells were cultured, induced with an adipogenic cocktail with or without Pio and cell numbers determined after 8 days of differentiation (Figure 3C). Without Pio, there was no difference in cell number of the WT, sh-GFP and sh-St2 adipocytes. However, the sh-St1 cell growth was retarded by 40–50% compared with sh-GFP cells, suggesting that the mitotic clonal expansion phase of 3T3-L1 adipogenesis is blocked upon *Stamp1* loss [7]. Differentiation in the presence of Pio did not affect cell growth at day 8 of differentiation for WT, sh-GFP and sh-St2 cells. In contrast, Pio partially rescued the cell number defect in sh-St1 cells with an increase from 40–50% to 65% compared to that of sh-GFP cells. This partial rescue was already maximal with 1 µM Pio and did not increase further up to 8 µM (Figure 3D). Taken together, these data show that *Stamp1* or *Stamp2* knockdown inhibits adipogenesis, and in the case of *Stamp1*, this may be, at least in part, through disruption of the mitotic clonal expansion in 3T3L1 cells.

### *Stamp1* and *Stamp2* Knockdown Affects *Stamp* Expression in 3T3-L1 Adipocytes

**Figure 4:**
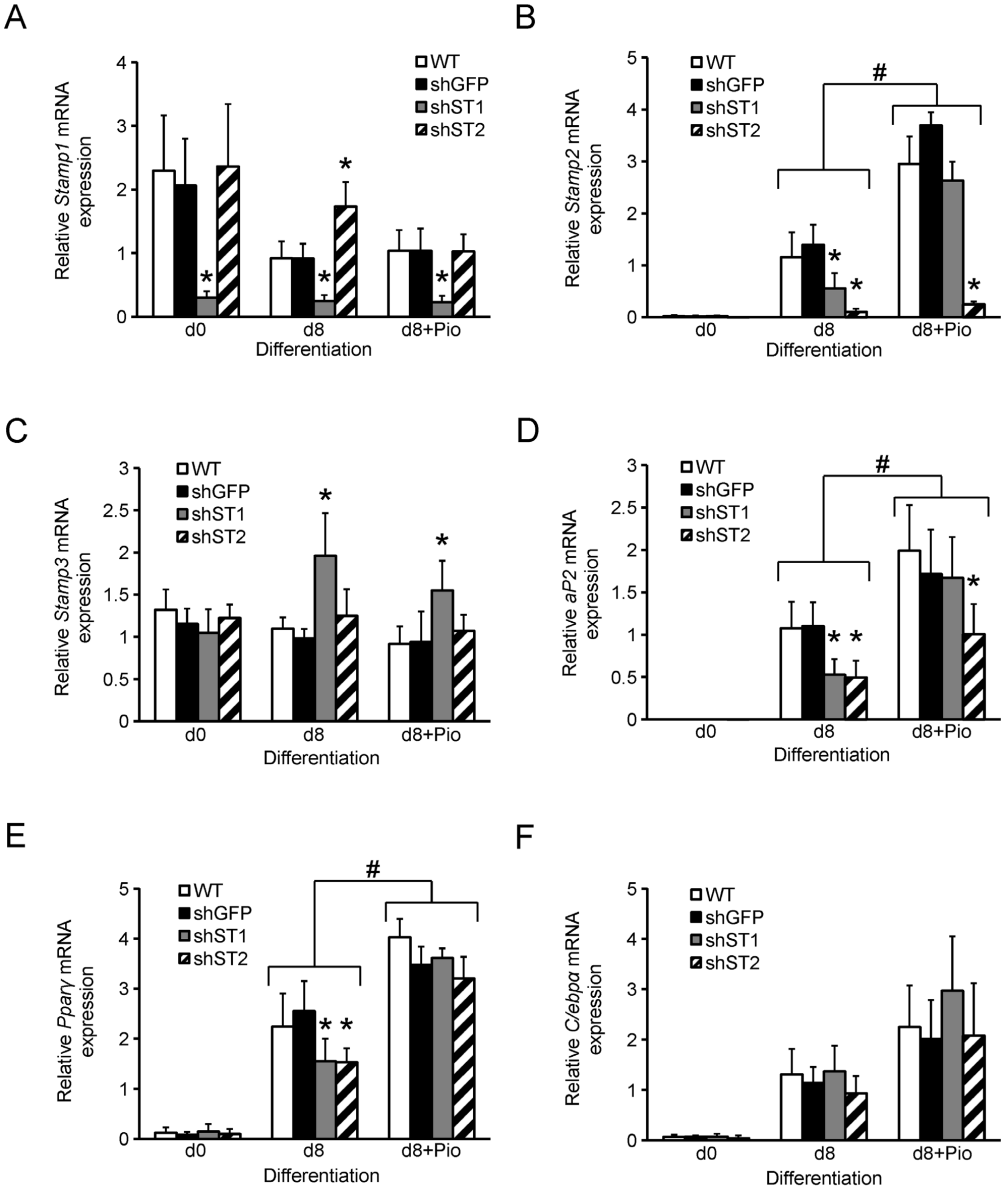
*Stamp1* or *Stamp2* knockdown reduces *Pparγ* and *aP2*, but not *C/ebpα*, mRNA expression in 3T3-L1 adipocytes. (A–F) qRT-PCR analysis of WT, sh-GFP, sh-St1 and sh-St2 cells harvested at day 0 (d0) and day 8 of differentiation with (d8+Pio) or without (d8) Pio. The figures show the relative mRNA expression of *Stamp1* (A), *Stamp2* (B), *Stamp3* (C), *aP2* (D), *Pparγ* (E), and *C/ebpα* (F) normalized to the reference gene *36B4* from three independent experiments, n = 9. *p<0.05 compared to sh-GFP cells; #p<0.05 between brackets.

As presented above, all *Stamps* are expressed and regulated during 3T3-L1 adipogenesis (Figure 1). To assess whether *Stamp1* or *Stamp2* knockdown influences expression of other *Stamps*, we determined *Stamp* expression in WT, sh-GFP, sh-St1 and sh-St2 cells at different time points during adipogenesis. As previously observed, *Stamp1* expression was decreased by 85% in sh-St1 cells, compared to WT and sh-GFP cells at day 0 (Figure 4A). At day 8, *Stamp1* levels were 60% lower in WT and sh-GFP cells compared with the same cells at day 0, consistent with Figure 1A, and remained low in sh-St1 cells. Interestingly, in sh-St2 cells, *Stamp1* expression increased by about 2-fold at day 8 of differentiation compared to WT and sh-GFP cells, but this was lost upon Pio treatment suggesting that Pparγ may inhibit *Stamp1* expression in these cells.

*Stamp2* expression was low at day 0 and increased dramatically in WT and sh-GFP cells at day 8 of differentiation (Figure 4B). Consistent with Figure 2B, *Stamp2* expression was decreased by 90% in sh-St2 cells. Interestingly, at day 8, *Stamp2* expression in sh-St1 cells was reduced by 60% compared to WT and sh-GFP cells. In the presence of Pio, *Stamp2* expression in WT, sh-GFP and sh-St2 cells increased by about 2.5-fold compared to levels at day 8 in the absence of Pio. In contrast, *Stamp2* levels in the sh-St1 cells rose by nearly 5-fold to reach comparable levels of WT and sh-GFP cells in response to Pio. These data suggest that Pparγ activation in increases *Stamp2* expression.

We also assessed *Stamp3* levels and observed no change in expression under similar conditions (Figure 4C), except for in sh-St1 cells where *Stamp3* expression was 2-fold higher at day 8 and 65% higher at day 8+ Pio compared with at day 0. These data suggest that Stamp1 and Stamp3 may have overlapping roles in 3T3-L1 adipocytes.

### *Stamp1* or *Stamp2* Knockdown Interferes with Adipogenic Gene Expression

The data presented above showed that *Stamp1* or *Stamp2* knockdown suppresses 3T3-L1 differentiation (Figures 3A and 3B). We thus investigated whether the expression of a common adipogenic marker, aP2, and the two main transcription factors regulating adipogenesis, Pparγ and C/ebpα [36], were affected by *Stamp* knockdown. We first determined mRNA expression of *aP2*, *Pparγ* and *C/ebpα* in WT, sh-GFP, sh-St1 and sh-St2 cells at different time points during adipogenesis. *aP2* expression was not detected at day 0 of differentiation (Figure 4D), but was present at day 8 for all cell lines. Consistent with the observed reduction in differentiation of sh-St1 and sh-St2 cells presented above (Figures 3A and 3B), *aP2* expression was 50% lower in these cells compared to WT and sh-GFP cells. At day 8+ Pio, *aP2* expression increased significantly in all cell types, and its levels in sh-St1 cells were now similar to that found in the WT or sh-GFP cells, in agreement with the rescue effect of Pio on the differentiation of sh-St1 cells (Figures 3A and 3B). Surprisingly, although *aP2* expression in sh-St2 cells was 2-fold upregulated at day 8+ Pio compared to day 8, the *aP2* mRNA levels were still 40% lower at day 8+ Pio compared to sh-GFP cells. This suggests that aP2 levels generally correlated with differentiation properties of the different cells lines, except for in sh-St2 cells.

There was no significant difference in the *Pparγ* expression at day 0 of differentiation among the different cell lines (Figure 4E). At day 8, *Pparγ* expression in WT and sh-GFP cells increased by about 20-fold compared with that observed at day 0, consistent with previous findings [37]. In agreement with the reduction in adipogenesis seen in Figures 3A and 3B, the sh-St1 and sh-St2 cells had 40% less *Pparγ* expression compared to sh-GFP cells. Furthermore, consistent with the rescue effect on 3T3-L1 differentiation with Pio presented above, there was an overall upregulation in *Pparγ* expression at day 8+ Pio, where *Pparγ* expression in sh-St1 and sh-St2 cells was comparable to that in sh-GFP cells. These data show that *Pparγ* levels correlated well with the differentiation properties of the different cell lines.

Similar to *Pparγ, C/ebpα* expression was low for all cell lines at day 0 of differentiation and increased by about 20-fold by day 8 (Figure 4F). However, unlike *Pparγ* expression, all cell lines displayed comparable *C/ebpα* expression at day 8. At day 8+ Pio, there was a trend towards increased *C/ebpα* expression for all cell lines. These data suggest that *Stamp1* or *Stamp2* knockdown do not significantly affect *C/ebpα* expression.

**Figure 5:**
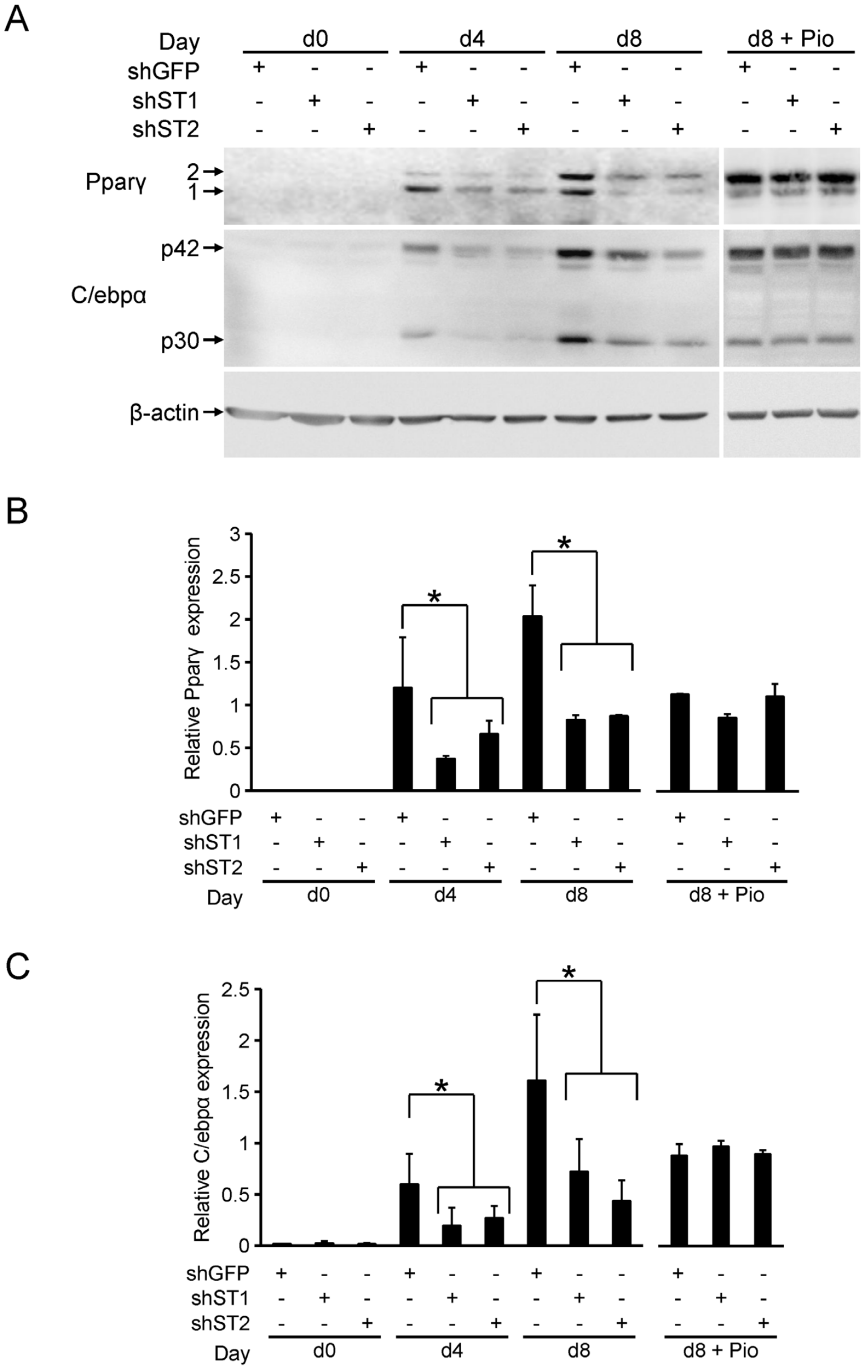
*Stamp1* or *Stamp2* knockdown reduces Pparγ and C/ebpα protein expression in 3T3-L1 adipocytes. (A) Western analysis showing Pparγ, C/ebpα and β-actin protein levels at day 0 (d0), day 4 (d4) and day 8 (d8) of differentiation in WT, sh-GFP, sh-St1 and sh-St2 cells, plus cells at day 8 differentiated with Pio (d8+ Pio). The data presented are representative of two independent experiments. (B–C) Quantification of Westerns in (A) with relative Pparγ (B) and C/ebpα (C) protein levels normalized to β-actin from two independent experiments, n = 6. *p<0.05.

We next investigated Pparγ and C/ebpα protein expression in the sh-GFP, sh-St1 and sh-St2 cells. These were harvested and cell extracts were made at different time points during adipogenesis and subjected to Western analysis. As shown in Figures 5A and 5B, Pparγ expression was not detectable at day 0, consistent with Figure 4E and previous reports [37]. By day 4 of differentiation, expression of both isoforms 1 and 2 of Pparγ were detected. In agreement with the observed mRNA expression (Figure 4E), Pparγ protein was expressed at 70% and 50% lower levels (for both isoforms) in the sh-St1 and sh-St2 cells, respectively, compared with sh-GFP cells. This decrease in expression was also present at day 8 of differentiation as both sh-St1 and sh-St2 cells had a 60% reduction in Pparγ expression compared to sh-GFP cells. Consistent with the mRNA levels presented in Figure 4E, differentiation with Pio resulted in similar Pparγ levels in both the control and knockdown cells (Figures 5A and 5B).

Similarly, C/ebpα expression was low at day 0 and increased 40-fold when the sh-GFP cells reached day 4 of differentiation, consistent with the data in Figure 4F. However, at day 4, the C/ebpα protein levels were 70% and 60% lower in the sh-St1 and sh-St2 cells, respectively, compared with sh-GFP cells. While C/ebpα expression continued to increase by 2.5-fold from day 4 to day 8 of differentiation in sh-GFP adipocytes, the expression in sh-St1 and sh-St2 cells remained 60% and 70% lower, respectively. Treatment with Pio rescued the loss in C/ebpα expression similar to what was seen for Pparγ (Figures 5A and 5C), but in contrast to the lack of changes observed at its mRNA level (Figure 3F). These data show that suppression of adipogenesis upon *Stamp1* or *Stamp2* knockdown is correlated to downregulation of Pparγ and C/ebpα protein expression.

### *Stamp1* or *Stamp2* Knockdown do not Affect C/ebpβ Expression and Nuclear Localization at the Early Stages of 3T3-L1 Adipogenesis

**Figure 6:**
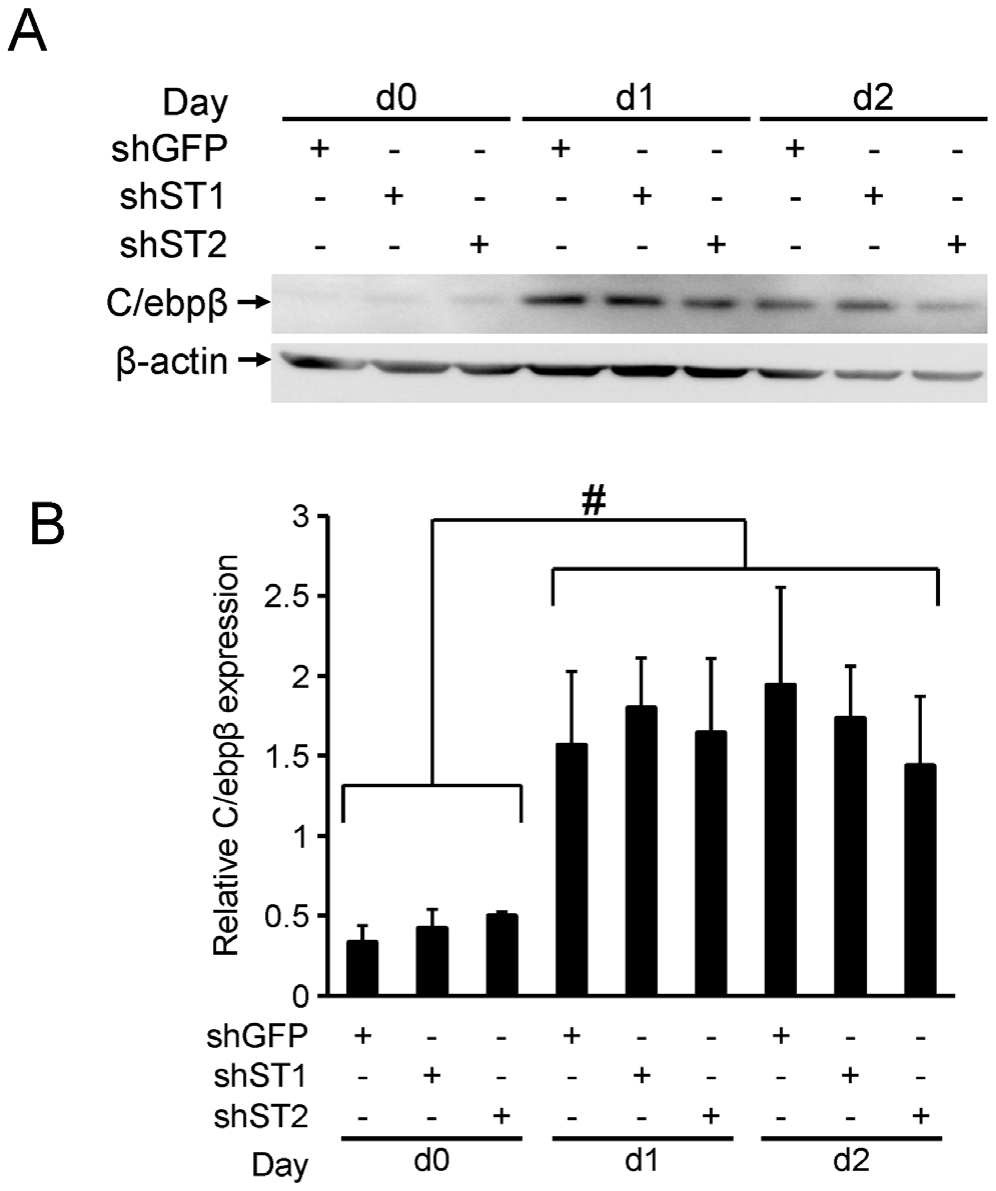
*Stamp1* or *Stamp2* knockdown does not affect C/ebpβ protein expression in early 3T3-L1 adipogenesis. (A) Western analysis showing C/ebpβ protein levels at day 0 (d0), day 1 (d1) and day 2 (d2) of differentiation with the same cells as in (5A). (B) Quantification of Western analysis results in (A) with relative C/ebpβ protein levels normalized to β-actin from two independent experiments, n = 6. #p<0.05 between brackets.

The transcription factor C/ebpβ is required for both Pparγ and C/ebpα expression early in adipogenesis [38]. We therefore examined whether there were alterations in C/ebpβ expression in early 3T3-L1 differentiation upon *Stamp1* or *Stamp2* knockdown. To that end, sh-GFP, sh-St1 and sh-St2 cells were harvested at days 0, 1 and 2 of differentiation and C/ebpβ expression was determined (Figure 6). Consistent with previous findings [39], C/ebpβ was expressed at low levels in sh-GFP cells at day 0, increased by approximately 3-fold by day 1, and remained unchanged at day 2. The sh-St1 and sh-St2 cells expressed C/ebpβ at comparable levels to the sh-GFP cells at all time points suggesting that C/ebpβ expression is not affected by Stamp1 or Stamp2.

C/ebpβ phosphorylation has been reported to induce its translocation to the centromeres of chromosomes, which has been linked to regulation of mitotic clonal expansion in 3T3-L1 adipogenesis [39]. We examined if this process was affected by *Stamp1* or *Stamp2* knockdown. WT, sh-GFP,sh-St1 and sh-St2 cells were differentiated and C/ebpβ localization was assessed with immunofluorescence confocal microscopy (Figure S1). In all cell lines, we observed the characteristic punctate localization of C/ebpβ [39]. These data suggest that C/ebpβ expression and localization are not affected by *Stamp1* and *Stamp2* knockdown.

### *Stamp2* Knockdown Reduces Superoxide Production in 3T3-L1 Cells Independent of Adipogenesis

**Figure 7:**
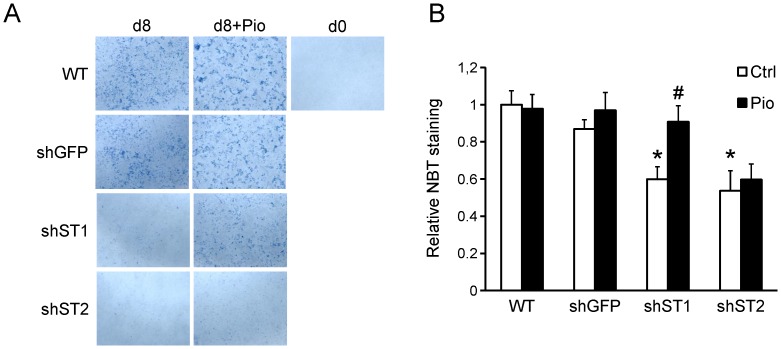
*Stamp2* knockdown reduces superoxide production in 3T3-L1 cells independent of adipocyte differentiation. (A–B) NBT assay with WT, sh-GFP, sh-St1 and sh-St2 cells differentiated with (Pio) or vehicle (Ctrl). (A) Representative images of the staining. (B) Quantification from three independent experiments, n = 9. *p<0.05 compared to sh-GFP cells; #p<0.05 between Ctrl and Pio groups.

When ectopically expressed in HEK293T cells, all Stamps demonstrate metalloreductase activity [18] which is predicted to be driven by NADPH oxidation. A byproduct of NADPH oxidation is generation of superoxide which has been implicated in adipogenesis [40]. To assess whether *Stamp1* or *Stamp2* knockdown affect superoxide production in adipocytes, we used the nitroblue tetrazolium (NBT) assay on WT, sh-GFP, sh-St1 and sh-St2 adipocytes [41]. There was a 40% and 50% reduction in the generation of superoxide in the sh-St1 and sh-St2 cells, respectively, compared to WT and sh-GFP cells (Figure 7). When the cells were differentiated with Pio, the superoxide production was unchanged for the WT, sh-GFP or sh-St2 cells. Interestingly, Pio significantly increased superoxide levels in sh-St1 cells which reached comparable levels to those in WT and sh-GFP cells. These data indicate that both Stamp1 and Stamp2 play a role in superoxide production in 3T3-L1 adipocytes and that the loss of superoxide levels upon Stamp1 knockdown can be rescued by Pparγ activation.

## Discussion

In this study, we examined the expression and possible function of Stamp family members during adipogenic differentiation. Previous work has shown that Stamp2 mRNA and protein expression is upregulated during adipogenesis [20], [21]. Stamp3 protein expression was also found upregulated by 75% in 3T3-L1 adipocytes compared to confluent fibroblasts [30]. Here we show that Stamp1 and Stamp3 mRNA expression are also differentially regulated during adipogenic conversion. These data, along with the effects of Stamp1 and Stamp2 knockdown on adipogenic conversion that we present, suggest that the Stamp family has a significant role in regulating adipogenesis.

Stamp1 knockdown significantly reduced 3T3-L1 differentiation (Figures 3A and 3B) which was unexpected as *Stamp1* was clearly downregulated upon adipogenic conversion (Figure 1A). Assessment of the number of adipocytes revealed that the sh-St1 cells displayed stunted proliferation upon induction of differentiation compared to the WT and sh-GFP cells suggesting that *Stamp1* knockdown interfered with the mitotic clonal expansion (Figures 3C and 3D). The mechanism(s) through which Stamp1 may regulate mitotic clonal expansion is currently not known.

In contrast to sh-St1 cells, sh-St2 cells proliferated at the same rate as the WT and sh-GFP cells during differentiation (Figure 3C) indicating that the mechanisms through which Stamp1 and Stamp2 regulate adipogenesis is different. Furthermore, there may be differences in the functioning and regulation of the mouse and human STAMP2 proteins. Inhibition of STAMP2 in human preadipocytes using an antibody, or STAMP2 knockdown prior to differentiation, did not affect adipogenesis [24], [42]. In contrast, there were clear inhibitory effects of Stamp2 knockdown in 3T3-L1 cells (Figure 3A and 3B). However, it is important to note that in the studies concerning Stamp2 in human preadipocytes, the thiazolidinedione (TZD) rosiglitazone (Rosi), a Pparγ activator, was used to induce adipogenesis, rather than the regular differentiation cocktail [31]. This could be, at least in part, the reason for these differential findings.

Here, we also show that both TZDs, Rosi and Pio, counteracted the inhibitory effect of *Stamp2* knockdown on 3T3-L1 cell differentiation (Figures 3A, 3B and S2). These data support the notion that Pparγ activation can circumvent *Stamp2* knockdown-mediated deficiency in adipogenesis in both mouse and human cells. In agreement with the inhibitory effects of *Stamp1* and *Stamp2* knockdown on 3T3-L1 adipogenesis, Pparγ expression was inhibited in both the sh-St1 and sh-St2 cells (Figures 4E, 5A and 5B). Interestingly, *C/ebpα* mRNA expression was similar in sh-St1 and sh-St2 cells compared to WT and sh-GFP cells, but C/ebpα protein levels were downregulated (Figures 4F, 5A and 5C); this suggests that there may be posttranscriptional control mechanisms that regulate C/ebpα expression. Consistent with the effect of Pio on 3T3-L1 differentiation addressed above, the overall Pparγ and C/ebpα expression increased and the protein levels were restored in sh-St1 and sh-St2 cells at day 8+Pio.

Functional C/ebpβ expression and activity was previously shown to be essential for inducing Pparγ expression and for the mitotic clonal expansion during adipogenesis [39]. However, there was no difference in C/ebpβ expression and localization between sh-GFP, sh-St1 and sh-St2 cells (Figures 6 and S1). Consistent with the stunted growth of the sh-St1 cells, we have previously shown that STAMP1 knockdown in human prostate cancer cells decreases proliferation by deregulating cell cycle related protein expression and activation of the mitogen-activated protein kinase (MAPK) pathway [35]. Whether Stamp1 could be involved in regulation of similar events during 3T3-L1 adipogenesis would need to be addressed in future studies.

Increased superoxide production was previously reported during 3T3-L1 adipocyte maturation [40]. Consistently, sh-St1 and sh-St2 cells showed lower superoxide production than WT and sh-GFP cells (Figure 7), and the generation of superoxide was restored in sh-St1 cells when differentiated with Pio. However, in sh-St2 cells superoxide levels remained low even with Pio. This again supports the notion that Stamp1 and Stamp2 affect adipogenesis through different mechanisms. These data are also consistent with the function of the metalloreductase domain of Stamp2 that can reduce iron with NADPH as an electron donor generating superoxide in the process [18]. Recently, an increase in mammalian target of rapamycin complex 1 (mTORC-1) dependent mitochondrial complex III superoxide production has been found to be required for Pparγ expression during adipocyte differentiation [11]. It was also found that Forkhead box O 1 (FOXO1) contributes to regulating endogenous antioxidants along with the increased ROS production during adipogenesis as FOXO1 knockdown led to downregulation of antioxidants and decreased 3T3-L1 differentiation [43]. Interestingly, in Stamp2 knockout mice several endogenous antioxidants are also downregulated [21]. Together with our findings, these point to a role of Stamp2 as a direct activator of 3T3-L1 adipogenesis through production of pro-adipogenic ROS signals. Another possibility is that Stamp2 may have a more indirect role in this process by contributing to antioxidant production that protects adipocytes from increasing ROS levels during adipogenesis. If Stamp2 levels are decreased, this will result in lower antioxidant production during differentiation, which could force the cells to adapt to lower ROS levels that can in turn retard the adipogenic conversion. In support of the latter, increased ROS and decreased insulin response was seen when STAMP2 was inhibited with an antibody in mature human adipocytes [44].

In summary, our data suggest that the Stamp family may have important roles in 3T3-L1 adipogenesis, which may involve distinct pathways for the different Stamps. These data pave the way to further explore the functions of the Stamp family in adipogenesis.

## Supporting Information

Figure S1 *Stamp1* or *Stamp2* knockdown does not affect C/ebpβ nuclear distribution in 3T3-L1 cells after induction of differentiation. (A–B) Immunofluorescence confocal microscopy analysis of expressed C/ebpβ (green) in the nuclei (blue) of WT, sh-GFP, sh-St1 and sh-St2 cells 16 h after induction of differentiation. (A) Representative images of the staining from two independent experiments. (B) Quantification from one experiment, n = 100.
(TIF)

Figure S2 Rosiglitazone reverses *Stamp1* or *Stamp2* knockdown-induced decrease in adipogenesis. Oil Red O staining of WT, sh-GFP, sh-St1 and sh-St2 cells differentiated with rosiglitazone (Rosi) or vehicle (Ctrl). Figure shows quantification from one experiment, n = 3. *p<0.05 compared to sh-GFP cells; #p<0.05 between Ctrl and Rosi groups.
(TIF)
