# Supplementary figures and images for: Differential Expression and Function of Stamp Family Proteins in Adipocyte Differentiation
Source: PLoS One. 2013 Jul 10;8(7):e68249. doi: 10.1371/journal.pone.0068249 (PMC3707909; doi:10.1371/journal.pone.0068249)

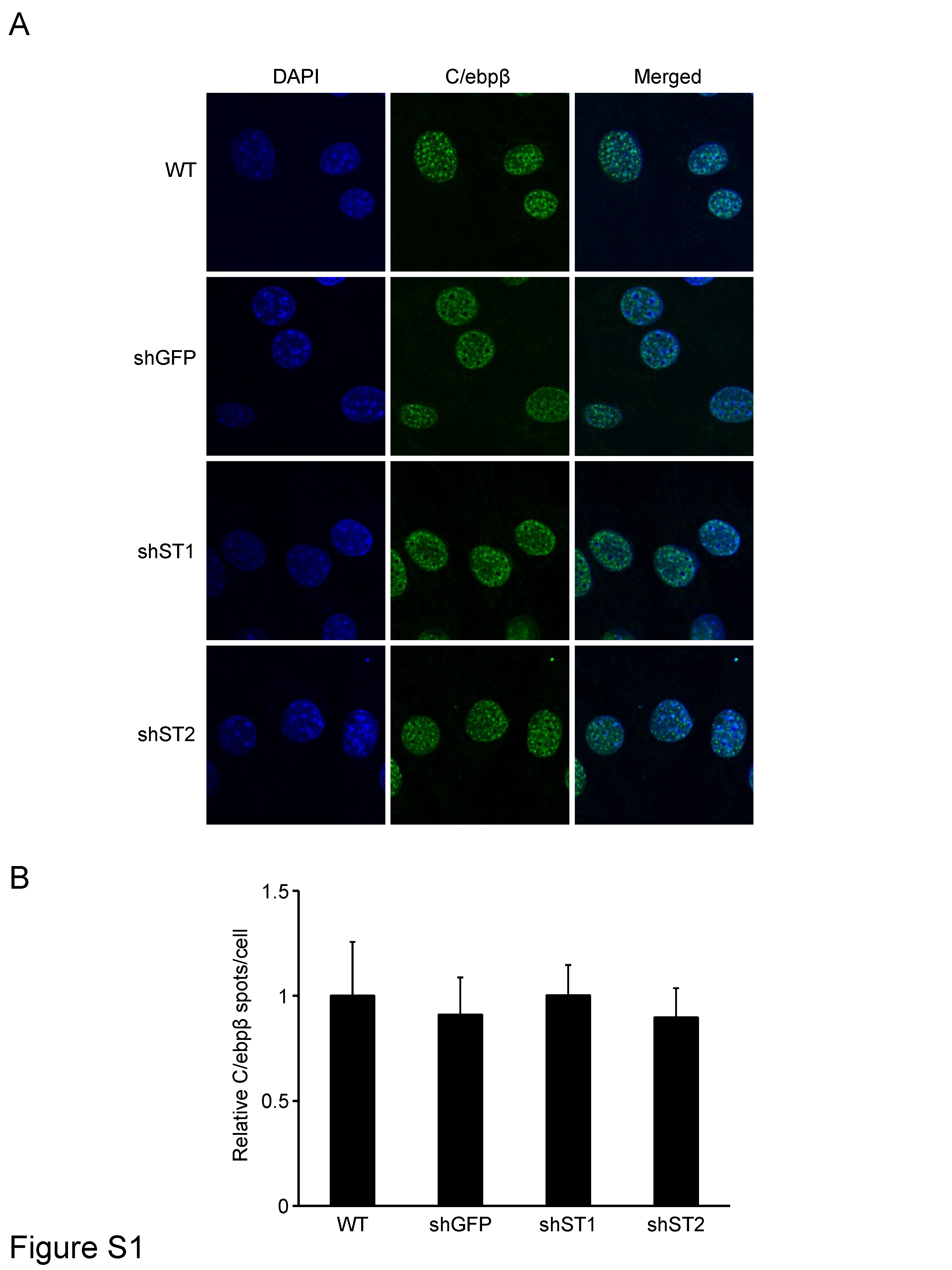

Supplement: Figure S1 — Stamp1 or Stamp2 knockdown does not affect C/ebpβ nuclear distribution in 3T3-L1 cells after induction of differentiation. (A–B) Immunofluorescence confocal microscopy analysis of expressed C/ebpβ (green) in the nuclei (blue) of WT, sh-GFP, sh-St1 and sh-St2 cells 16 h after induction of differentiation. (A) Representative images of the staining from two independent experiments. (B) Quantification from one experiment, n = 100. (TIF) [file pone.0068249.s001.tif]
